# Supplementary material for: Learning curve and surgical outcome of robotic assisted colorectal surgery with ERAS program
Source: Sci Rep. 2022 Nov 29;12:20566. doi: 10.1038/s41598-022-24665-w (PMC9709162; doi:10.1038/s41598-022-24665-w)
Supplement: Supplementary file 1 — Supplementary Information 1. [file 41598_2022_24665_MOESM1_ESM.zip › LSC Raw data-Ñ[▒K/108/H_ñw╝╨░O▒Kñσ.pdf]

正式摘要(電子簽章：000000205236768)

W86 / W86 英文標題(注意:無英文姓名-請補病人基本檔!)

【讀取】憑證

臺 中 榮 民 總 醫 院

出院病歷摘要

轉入號：　　　　　　轉出號：

[redacted]

(8)流水編號	
(9)入院日期	109/12/08 (2020) 直腸外科 W86 - 17
(10)轉科日期	
(11)出院日期	109/12/14 (2020) 住院天數計 6 日

診斷

- 入院:Rectal cancer
  Hypertension
- 出院:Rectal cancer s/p laparoscopic LAR with colo-anal anastomosis on 2020/12/9
  Hypertension

主訴

- no special discomfort

病史

- This 65 years old male patient has past history of hypertension. He recieved regular health exam and colon polyp was found. Thus, he came to our OPD for help. Colonoscopy revealed multiple polyp at 35, 25, 20, 15, 10cm from anal verge and biopsy was done. The result showed adenocarcinoma (10cm FAV) with submucosa invasion. Thus, he was admitted for surgical treatment.

體檢發現

- Anal inspection:
  Recto digital examination:
  Sigmoidoscopy:
  Anoscopy:
  Abdomen:
  2020/11/26 3F sedative colonoscopy up to cecum smoothly, normal mucosa, much polyps from rectum to 35cm FAV, s/p hot snare polypectomy + clipx1 at 35cm, 25cm, 20cm, s/p hot snare polypectomy at 15cm, s/p EMR + clipx1 at 10 cm FAV, need follow up 1 year
  - General appearance :
    - easy looking , conscious clear , without cardiopulmonary distress
    - Vital signs自動帶入date 體溫(BT) 脈搏(P) 呼吸(R) 血壓(BP) 身高(BH) 體重(BW) BMI
      20201208 36.2℃ 69 次/分 18 次/分 147/86mmHg 176cm 83kg 26.8
  - HEENT :
    - Eyes: no anemic conjunctiva, no icteric sclera, normal EOM
    - ENT: no otorrhea, no rhinorrhea, no erythematous mucosa, no injected tonsil, no deformity
  - Neck: supple, freely movable, trachea midline,no deformity, no engorged jugular vein , no palpable thyroid or lymph node enlargement
  - Chest:
    - Inspection: Grossly normal, symmetric and fully expansion, no accessory muscle used, no operation scar, no spider angiomata, no gynecomastia
    - Palpation: no palpable mass or nodule, no subcutaneous emphysema, no tenderness
    - Ausculation: clear breathing sound
  - Heart:
    - Percussion: normal heart size
    - Ausculation: regular heart beat, normal S1, S2, no gallop, no murmur
  - Abdomen:
    - Inspection: in
      - flat contour, symetrically, no scar, no stria, no dilated veins, no rashes
    - Ausculation: normoactive bowel sound, no abdominal bruits
    - palpation: soft, no muscle guarding or rigidity, no local tenderness, no rebounding pain, no palpable mass, no palpable pulsation
    - Percussion: no shifting dulllness, no hepatomegaly, no splenomagaly
  -

Peripheral pulsation :	CA	BA	RA	FA	PA	PTA	DPA
Right side :	++	++	++	++	++	++	++/+++
Left side :	++	++	++	++	++	++	++/+++

  - Genito-anus: no tenderness or palpable mass lesion or rectal shelf, normal external genitalia, normal anal tone, brown stool

CRS CONDITION

- Colonoscope report: much polyps from rectum to 35cm FAV, s/p hot snare polypectomy + clipx1 at 35cm, 25cm, 20cm, s/p hot snare polypectomy at 15cm, s/p EMR + clipx1 at 10 cm FAV, need follow up 1 year
- CEA: 1.5 ng/ml
- Imaging studies(Sono , CT of abdomen and chest Films):
  - 檢驗項目:SONO abdomen 報告日期:1091120
    主 訴:
    Result: Finding: > Mild fatty liver change. > Left renal stone about 1.1cm. > Otherwise, no other specific findings in this study. Impression: As above description.
-
  - Lung

手術日期及方法

- 手術日期及方法20201209 1. Laparoscopic low anterior resection, side-to-end anastomosis, EndoGIA 60/.38mm, CEEA- 29mm. Bilateral doughnut: intact, air tight test: done, no air leak.
  2. High ligation (+) above the left colic artery.
  3. Cut margin: proximal 15 cm, distal 3 cm.
  5. Take down of splenic flexure: (-).
  6. Intraoperative colonoscopy done for check post polypectomy scar
  7. A 12mm penrose drain placed in the pelvis

住院治療經過

- After admission, operation with laparoscopic low anterior resection was done on 12/9 smoothly as scheduled. Penrose drain was removed on POD3 due to less amount. Foley was removed on 12/13 but he still complained urinary difficulty so Foley was inserted back. Under the relatively stable condition, he was discharged on 12/14.

合併症

- nil

一般檢查

- 累積報告：

DATE	TIME	ALB	TP	BIL.T	BIL.D	ALKP	AST	ALT	LDH	CHOL	TG	HDL-C	LDL	UA	NH3	AMY
1091210	0851						19	28								

DATE	TIME	WBC	RBC	HGB	HCT	MCV	PLT	BLST	NEUT	BAND	SEG	LYM	MON	EOS	BAS
1091210	0828	18660		12.0	34.2		157		90.2			6.8	2.9	0.0	0.1
1091214	0835	5770		12.4	35.4		161		64.5			24.3	6.4	4.5	0.3

DATE	TIME	NA	K	CL	CA	BUN	CREAT	Mg	P	CRP	ESR	PCT	LIPASE	LACTATE
1091210	0851	147	3.6		8.4	15	1.44							
1091214	0843	144	3.3		8.5	8	1.39							

特殊檢查

病理報告

其它

出院時情況

- 3.改門診治療

出院指示

藥名	劑量	劑量單位	用法	頻次	數量	數量單位	現況	天數	單位
$Biofermin tab	2	TAB	PO	TID	90	TAB	TH	15	天
Sevikar tab 5/20mg	1	TAB	PO	QD	15	TAB	TH	15	天
Ulstop tab 20mg	1	TAB	PO	BID&HS	45	TAB	TH	15	天
Ultracet tab	1	TAB	PO	BIDPRN	30	TAB	TH	15	天
Loperamide cap 2mg	1	CAP	PO	QDPRN	15	CAP	TH	15	天
Mag. Oxide tab	2	TAB	PO	TID	90	TAB	TH	15	天
OCAS Harnalidge 0.4 mg	1	TAB	PO	QDAC	15	TAB	TH	15	天

出院計劃

主治醫師_2062F 蔣鋒帆 / 住院醫師_2331F 陳凱祥

正式摘要(電子簽章：**000000205236768)**

W86 / W86

doctorRank:B
